# Theoretical Analysis and Characteristic Study of Li-Doped P-Type ZnO Ultra-Thin Cantilever Beam Accelerometer

**DOI:** 10.3390/ma18081766

**Published:** 2025-04-11

**Authors:** Yingqi Shang, Jiayu Bi, Weiwei Liu, Chunpeng Ai, Hongquan Zhang

**Affiliations:** 1 College of Electronic Engineering, Heilongjiang University, Harbin 150001, China; shangyingqi@163.com (Y.S.); aichunpeng@hlju.edu.cn (C.A.); 2 The 49th Research Institute, China Electronics Technology Group Corporation, Harbin 150001, China; bjy8866@163.com (J.B.); 2191277@s.hlju.edu.cn (W.L.)

**Keywords:** ZnO, piezoelectricity, cantilever beam

## Abstract

Nonlinear correction was performed on the mechanical motion of ultra-thin cantilever beams, and strain effects were calculated on ultra-thin multi-layer heterogeneous material stacked cantilever beams. The atomic structure and piezoelectric properties of ZnO were studied using first-principles calculations. In this study, generalized gradient approximations of Perdew–Burke–Erzerhof (GGA-PBE) functionals and Plain Wave Basis Sets were used to calculate the electronic structure, density of states, energy bands, charge density, and piezoelectric coefficient of intrinsic ZnO. Research and calculations were conducted on Li-doped ZnO with different ratios. According to our calculations, as the Li doping ratio increases from 0 to 10%, the bandgap width of ZnO material increases from 0.74 to 1.21 eV. The results for the density of states and partial density of states indicate that the increase in band gap is due to the movement of Zn-3d states towards the high-energy end, and the piezoelectric coefficient of the material increases from 2.07 to 3.3 C/m^2^. Meanwhile, based on the optimized Li-doped ZnO cantilever beam accelerometer, an ultra-thin cantilever beam accelerometer with a sensitivity of 7.04 mV/g was fabricated.

## 1. Introduction

With the development of the semiconductor industry, the development of third-generation semiconductors led by SiC, GaN, and ZnO is of great significance for promoting technological progress and industrial upgrading in fields such as energy, communication, and transportation. The flourishing development of MEMS technology has provided a powerful driving force for research on various microstructures, microsensors, and microactuators. As one of the common microstructures in MEMS, microcantilever beams have been widely used in various fields, such as physical quantity detection, biochemical sensing, environmental detection, etc., due to their advantages of low cost, light weight, low power consumption, small size, high sensitivity, and good response speed. There are various forms of cantilever beams, including rectangular, triangular, T-shaped, U-shaped, tuning-fork-shaped, bridge-shaped, etc. Different forms of microbeams are typically applied in different fields, with rectangular beams being the most widely used.

The working principle of an ultra-thin suspended-beam piezoelectric accelerometer can be simply described as when the piezoelectric accelerometer is at rest without external force, the upper and lower surfaces of the piezoelectric film are in a state where positive and negative charges coincide at the central axis of the film, and the entire film is in an electrically neutral state. When an external load is applied at the free end of the piezoelectric suspension beam, the suspension beam undergoes deformation and bending, and the piezoelectric film also undergoes deformation. The positive and negative charges inside the film separate, and a potential difference is generated between the positive and negative charges on both sides of the film. The charges are amplified and converted into voltage signals for output, achieving the measurement of acceleration signals. In recent years, the production of piezoelectric acceleration sensors has mainly involved the materials shown in Table 1, which details the sensitivity of the prepared acceleration sensors.

ZnO is a II–IV semiconductor material, and its stable phase at room temperature and pressure has a hexagonal wurtzite structure. It has a forbidden bandwidth (Eg = 3.37 eV), high exciton binding energy (60 meV), low dielectric constant, strong radiation resistance, good electromechanical coupling performance, high chemical and thermal stability, and excellent piezoelectric and optoelectronic properties. ZnO also has the advantages of non-toxicity, non-pollution, compatibility with EMS, easy availability of raw materials, and low cost. ZnO, with its unique, physical, chemical, and electronic properties, occupies an important position in the research and application of semiconductor materials, such as light-emitting devices, strain sensors, transparent conductive films, electronic transmission, and other fields. With the deepening of research, its application areas may continue to expand.

There have been many studies on doped ZnO, and the first-principles analysis of doped ZnO has also received much attention. Using first-principles calculations of Cu/Al co-doped ZnO, Ma et al. [1,2,3,4] studied the band structure, density of states, and light absorption properties of different Cu-doped ZnO films to improve their transparency. Li et al. [5] studied the effect of different lutetium doping levels on the optoelectronic properties of ZnO materials using first principles. Zhang Tao et al. [6] calculated the energy band, density of states, differential charge density, and piezoelectric properties of Mg-doped ZnO using first principles. Zhou Xun et al. [7,8,9,10,11,12] studied the lattice constant, electronic structure, dielectric function, and optical absorption coefficient of hexagonal wurtzite-type ZnO doped with transition metal ions using first principles.

However, the first principles of Li-doped ZnO have not been studied yet. In this paper, the electronic structure energy states, density of states, differential charge density, and piezoelectric coefficient of Li-doped ZnO are calculated using first principles, and Li-doped ZnO thin films are fabricated. At the same time, an optimized Li-doped ZnO ultra-thin cantilever beam accelerometer is fabricated.

## 2. Working Principle of Ultra-Thin Cantilever Beam Accelerometer

**Figure 1:**
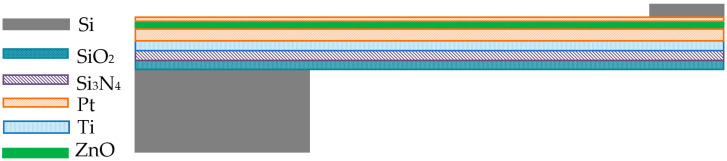
Structural diagram of ZnO ultra-thin cantilever beam accelerometer.

The focus of this study is the preparation of an accelerometer by sputtering ZnO piezoelectric thin film on an ultra-thin cantilever beam. The main layered structure shown in Figure 1, from bottom to top, consists of a SiO_2_ layer, Si_3_N_4_, Ti/Pt composite bottom electrode layer, ZnO piezoelectric thin film layer, and Pt top electrode layer, with amorphous silicon deposited as a mass block at the free end of the cantilever beam. Most piezoelectric thin-film microcantilever sensors adopt this structure. In the original cantilever beam structure, removing the Si beam and using SiO_2_ and Si_3_N_4_ as the substrate of the cantilever beam can effectively reduce the thickness of the beam film and improve the sensitivity of the piezoelectric accelerometer, but it also increases the difficulty of device preparation.

When the size of the material in the cantilever beam structure is reduced by a few micrometers, there will be significant differences in its physical, mechanical, and other material properties compared to when the materials in the cantilever beam structure are of macroscopic size. At this point, classical elastic theory cannot comprehensively analyze the elastic characteristics of cantilever beams, and size effects need to be supplemented into classical elastic theory. Therefore, it is necessary to revise the classical elasticity theory. The length of a cantilever beam is denoted as L. Under the action of a concentrated force P, the free end of the beam will move along the negative direction of the *x*-axis, and its displacement in the horizontal direction is denoted as Δx.

With strain *S* and electric displacement *E* as independent variables, the piezoelectric constitutive equation is

(1) $$D=-eS+\beta^{S}E$$

where *e* is the piezoelectric stress constant matrix.

$\beta^{S}$ is the inverse dielectric constant matrix under constant strain.

Rewrite the piezoelectric constitutive equation in this design as

(2) $$D=-e_{ij}S+\varepsilon_{ij}^{S}E$$

where $\varepsilon_{ij}^{S}$ is the inverse dielectric constant under constant strain.

When considering the strain gradient effect of ultra-thin cantilever beams, the constitutive equation of elastic materials can be rewritten as

(3) $$\sigma_{ij}=2\mu \varepsilon_{ij}+\lambda \delta_{ij}\varepsilon_{kk}-c\frac{\partial^{2}(2\mu \varepsilon_{ij}+\lambda \delta_{ij}\varepsilon_{kk})}{\partial m^{2}}$$

where

(4) $$\mu =\frac{E}{2(1+v)},\;\delta_{ij}=\left\{\begin{array}{c} 1,\;i=j;\\ 0,\;i\neq j. \end{array}\right.$$

c—Elastic gradient coefficient.

$\sigma_{ij}$—Stress tensor components.

$\varepsilon_{ij}$—Strain tensor components.

μ and λ—Coefficients of the material.

E—Elastic modulus.

v—Poisson’s ratio.

Assuming that the stress and strain of a cantilever beam only exist along the x-direction of σ and ε, the constitutive equation can be simplified as

(5) $$\sigma =E(\varepsilon -c\nabla^{2}\varepsilon )$$

where

(6) $$\nabla^{2}\left(\right)={\left(\right)}_{.mm}$$

Assuming the cross-section of a cantilever beam is rigid, the strain at any point on any cross-section of the cantilever beam is

(7) $$\varepsilon =\frac{y}{\rho }=y\theta \prime$$

Among these, ρ is the curvature radius at this point on the cantilever beam.

So, the cantilever beam has the following relationship:

(8) $$\left\{\begin{array}{c} \frac{dx}{ds}=\cos\theta \\ \frac{d\omega }{ds}=\sin\theta \end{array}\right.$$

**Figure 2:**
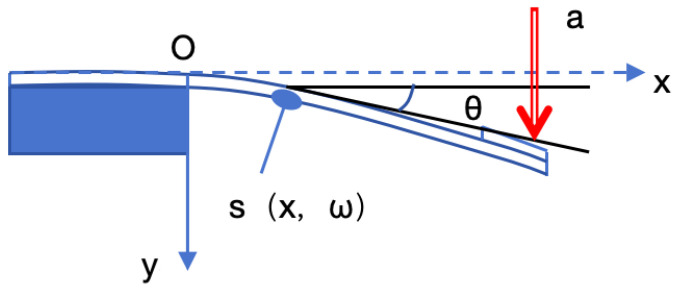
Deformation of ultra-thin cantilever beam.

As shown in Figure 2, the fixed end of the cantilever beam is located at x = 0:

(9) $$x\left(0\right)=0,\;w\left(0\right)=0,\;\theta \left(0\right)=0$$

At this point, the stress at any point on the cross-section of the cantilever beam is

(10) $$\sigma =E\left(\varepsilon -c\varepsilon^{\prime \prime }\right)=Ey(\theta^{\prime }-{c\theta }^{\prime \prime })$$

At this point, the strain at any point on the cross-section of the cantilever beam is

(11) $$U_{E}=\frac{1}{2}\int_{A}\int_{0}^{L}\sigma \varepsilon dsdA=\frac{1}{2}EI\int_{0}^{L}({\theta^{\prime }}^{2}-{c\theta }^{\prime \prime }\theta^{\prime \prime \prime })ds$$

A—Cross-sectional area.

I—Cross-sectional area.

For the entire cantilever beam system, the total potential energy is composed of two parts: the elastic strain energy of the cantilever beam itself and the potential energy caused by external loads. Therefore, it can be concluded that

(12) $$\Pi =\frac{1}{2}EI\int_{0}^{L}({\theta^{\prime }}^{2}-{c\theta }^{\prime }\theta^{\prime \prime \prime })ds-Pw\left(L\right)+\int_{0}^{L}\lambda (w^{\prime }-sin\theta )ds$$

Integrate the above equation as follows:

(13) δΠ=EI∫0Lθ′δθ′ds−PδwL−12EIc∫0Lθ′δθ‴+θ‴δθ′      +∫0Lδλw′−sinθ+λδw′−cos⁡θδθds      =EIθ′δθL0−EI∫0Lθ″δθds−PδwL      +∫0Lδλw′−sin⁡θds+λδwL0+EIc∫0Lθ4δθds      −∫0Lλcosθδθds−12EIcθ′δθ″L0+12EIcθ″δθ′L0      −[EIcθ‴δθ′]L0

According to the principle of minimum potential energy, δΠ = 0 is obtained.

(14) λ=P

(15) $${c\theta }^{\left(4\right)}-\theta^{\prime \prime }-\frac{\lambda }{EI}\cos\theta =0$$

(16) $$\theta^{\prime }\left(L\right)-c\theta^{\prime \prime \prime }\left(L\right)=0$$

(17) $$\theta^{\prime \prime }\left(L\right)\delta \theta^{\prime }\left(L\right)-\theta^{\prime \prime }\left(0\right)\delta \theta^{\prime }\left(0\right)-\theta^{\prime }\left(L\right)\delta \theta^{\prime \prime }\left(L\right)+\theta^{\prime }\left(0\right)\delta \theta^{\prime \prime }\left(0\right)=0$$

Due to the non-zero curvature of the fixed end of the cantilever beam, according to the boundary conditions, the constant displacement differential equation of the cantilever beam can be rewritten as

(18) $$\left\{\begin{array}{c} {c\theta }^{\left(4\right)}-\theta^{\prime \prime }-\frac{P}{EI}\cos\theta =0\\ x^{\prime }=\cos\theta \\ w^{\prime }=\sin\theta \end{array}\right.$$

When the deformation of the cantilever beam is small, the above equation is

(19) $$\left\{\begin{array}{c} {cw}^{\left(5\right)}-w^{\prime \prime \prime }-\frac{P}{EI}=0\\ dx=ds\\ w^{\prime }=\sin\theta \end{array}\right.$$

At this point, the changes before and after the deformation of the cantilever beam can be ignored.

Therefore, the deflection of the cantilever beam can be rewritten as

(20) $$w=\frac{P}{EI}L^{3}\left(\frac{\xi^{2}}{2}-\frac{\xi^{3}}{6}-\beta^{3}\frac{\sinh\left[\left(1-\xi \right)/\beta \right]}{\cosh(1/\beta )}+\beta^{3}\tanh\left(1/\beta \right)-\beta^{2}\xi \right)$$

where

(21) $$\xi =x/L,\beta =\sqrt{c}/L$$

The deformation angle of the cantilever beam is

(22) $$\theta =w^{\prime }=\frac{P}{EI}L^{2}\left(\xi -\frac{1}{2}\xi^{2}+\beta^{2}\frac{\cosh\left[\left(1-\xi \right)/\beta \right]}{\cosh(1/\beta )}-\beta^{2}\right)$$

It can be seen that the deformation angle and deflection of the cantilever beam are nonlinear. The larger the strain gradient, the smaller the deformation of the beam. When the strain gradient increases to a certain value, the gradient effect on the deformation of the beam will weaken. Therefore, when designing the size of a cantilever beam, the influence of the size of the cantilever beam on the strain gradient should be considered.

## 3. Construction of Li-Doped ZnO Model

**Figure 3:**
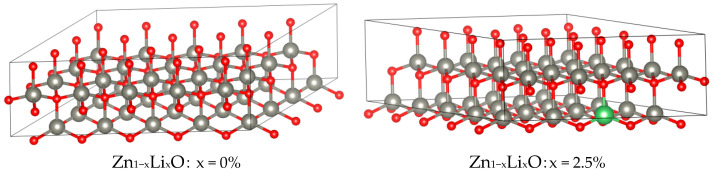
Atomic structure diagram. (The green is Li, the gray is Zn, and the red is O).

This article adopts a first-principles approach and uses GGA-PBE functionals and Plain Wave Basis Sets for calculations. Based on VASP 6.3.2 software, the truncation energy of the plane wave is set to 450 eV, and the force convergence standard and energy convergence standard are set to −10^−4^ eV/Å and 10^−8^ eV, respectively. When calculating the piezoelectric matrix, the density functional perturbation method is used for calculation. During modeling, ZnO with a hexagonal wurtzite structure (P63MM) was used as the unit cell, and the original cell of ZnO with the space group P63MM was expanded into a 5 × 4 × 1 supercell, as shown in Figure 3.

**Figure 4:**
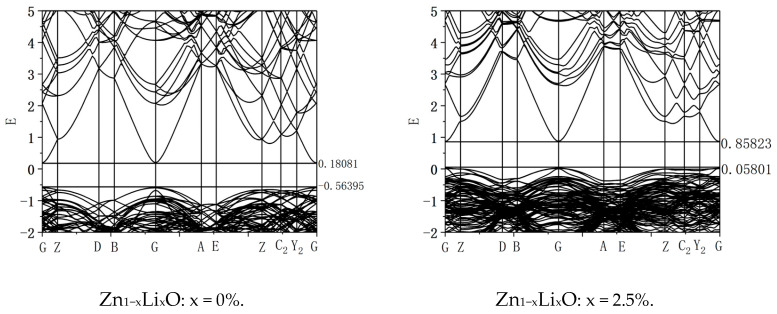
Energy band diagram.

**Figure 5:**
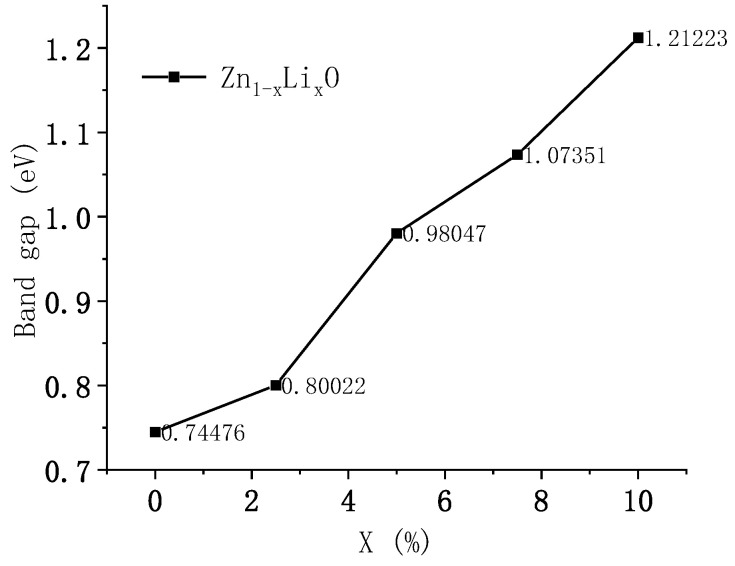
Relationship curve between Li doping amount and bandgap variation.

The energy band of Li-doped ZnO was calculated. Figure 4 shows the band diagram of ZnO, which shows that the conduction band bottom and valence band top are located at G. In the same position, during the electronic transition process, electrons can transition from the valence band top to the conduction band without changing the wave vector, indicating that ZnO is a direct bandgap semiconductor. The bottom of its conduction band has a value of 0.18081 eV, and the top of its valence band has a value of −0.56395 eV. The bandgap width has a value of 0.74476 eV. Zhang et al. [6] studied the electronic structure of ZnO using first principles and calculated the bandgap width of intrinsic ZnO to be 0.729 eV, which is consistent with the results of this study. The figure shows the band diagram with doping ratios of 0% and 2.5%. From the calculation results, it can be seen that as the doping ratio increases, the energy band shifts upward, the bottom of the conduction band moves up by 0.67 eV, and the top of the valence band moves up by 0.62 eV. The upward shift of the conduction band bottom is greater than that of the valence band top, and the bandgap width increases by 0.05 eV. Adjusting the doping ratio can alter the electronic structure of ZnO materials and adjust their bandgap width. Figure 5 shows the variation curve of Li doping concentration and bandgap width.

We then calculated the density of states of Li-doped ZnO. The density of states is the projection of energy states, and in regions with dense energy bands, the density of states is higher. In the energy region with sparse energy bands, the density is too low. In an energy range without band distribution, the density of states is equal to 0.

**Figure 6:**
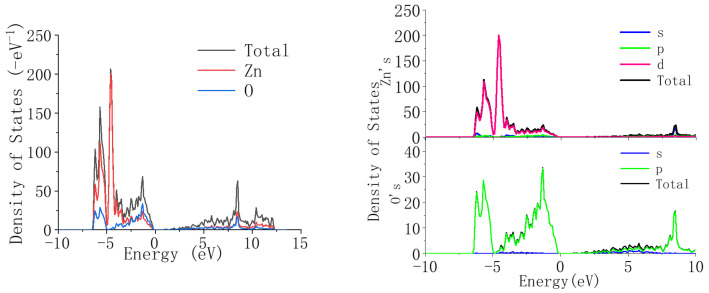
Density of states and partial density of states of ZnO.

Figure 6 shows the density of states of ZnO. It can be seen from the figure that in the valence band region between −10 eV and 0, the density of states of ZnO mainly constitutes the Zn-3d state and the O-2p state, and the two states coincide. The conduction band region mainly constitutes the Zn-4s state and 0-2p state, and the two states coincide.

**Figure 7:**
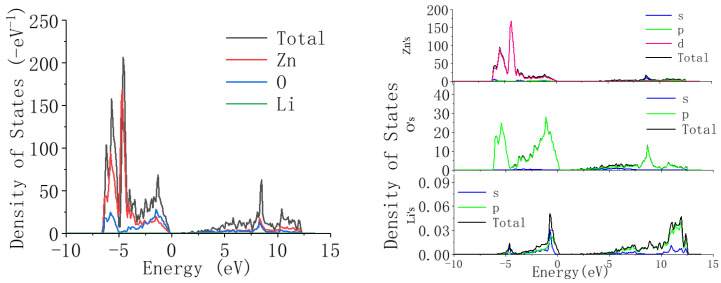
Zn_1−x_Li_x_O: density of states and partial density of states at x = 2.5%.

From Figure 6 and Figure 7, it can be seen that compared with undoped ZnO, when Li ions are introduced into ZnO, the position of the valence band does not change significantly, but the energy decreases. This is because the introduction of Li reduces the contribution of the Zn-3d state, but the contribution of the O-2p state does not change in detail, indicating that the valence band is mainly related to the O-2p state. Compared to the valence band, when Li ions are introduced into ZnO, the position of the conduction band shifts towards the high-energy region. This is because as the concentration of Li ions increases, the contribution of the Li-2p state gradually increases, and its high-energy contribution range is within 10–15 eV, thus causing the conduction band of ZnO to shift towards the high-energy region. From the above analysis results, it can be concluded that the introduction of Li can widen the bandgap of ZnO.

**Figure 8:**
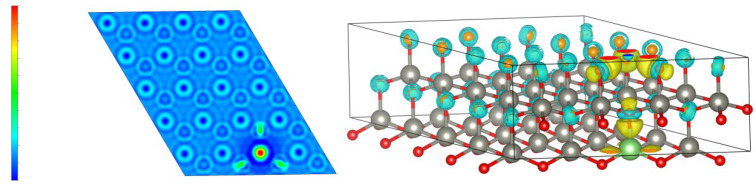
Differential charge density distribution of Zn_1−x_Li_x_O: x = 2.5% Li-doped ZnO.

Figure 8 shows the differential charge density plot, which reflects the charge distribution of ZnO after Li atom doping. When Li is doped into ZnO, it replaces Zn atoms and changes the charge distribution. The red color in the left figure indicates an increase in charge density, while the blue color indicates a decrease in charge density. The yellow charge density in the right figure increases, while the blue represents a decrease in charge density.

From the differential charge density plot, it can be concluded that when Li is introduced, the charge density distribution of ZnO changes, with an increase in charge density around Li atoms. As the proportion of Li doping increases, the amount of adsorbed charge around Li also increases accordingly. This is because Li has stronger metallicity than Zn and has a stronger ability to adsorb O, causing O to shift from Zn in the Li direction. The bonding energy between Li and O is enhanced, causing the bonding energy between Zn and O to decrease. The role of Li becomes increasingly evident, which is also the reason why the conduction band of Li-doped ZnO moves in the higher-energy direction.

We calculated the piezoelectric constant of Li-doped ZnO, where the piezoelectric constant is the conversion coefficient by which the piezoelectric material converts mechanical energy into electrical energy or vice versa. When conducting first-principles calculations, the calculation of e33 is usually performed. This represents the ratio of stress change to electric field strength change when a piezoelectric material is subjected to constant strain. The piezoelectric coefficient e33 represents the amount of charge change per unit volume under unit mechanical stress, and its physical meaning is to describe the strength of the piezoelectric effect. The larger the piezoelectric coefficient, the greater the change in charge generated by the material after being subjected to mechanical stress, indicating better piezoelectric performance of the material. The piezoelectric coefficients of ZnO with five different doping ratios were calculated, and the results are shown in the following Table 2.

Due to their hexagonal wurtzite structure, ZnO materials are prone to charge separation when pressure is applied along the *c*-axis. The lattice constant in the *c*-axis direction gradually decreases, resulting in a decrease in strain in the c-axis direction. e33 = ∂ P3/∂ ε, where ε is the strain in the *c*-axis direction. Since ε is inversely proportional to e33, its e33 value is relatively high.

**Figure 9:**
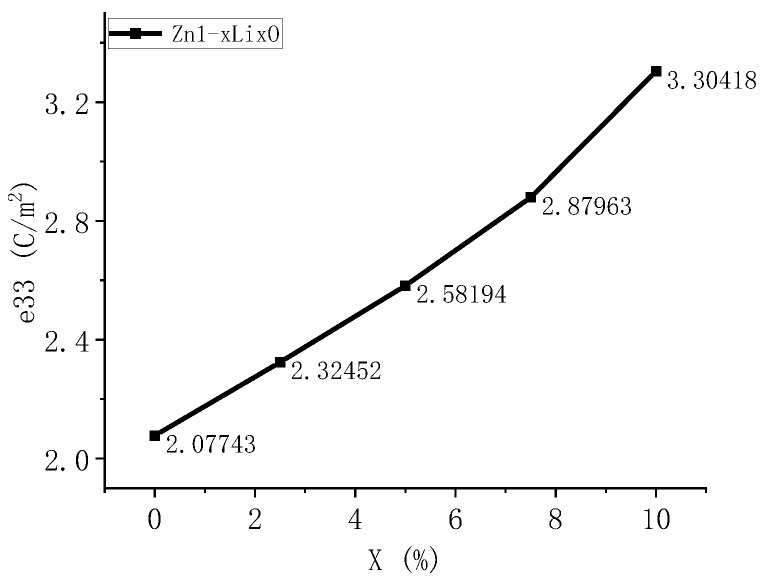
Changes in piezoelectric coefficient of ZnO doped with Li at different ratios.

The calculation results in Figure 9 show that when Li enters ZnO, the piezoelectric coefficient changes. As the concentration of Li increases, the piezoelectric coefficient also increases. This indicates that the introduction of Li into the ZnO lattice significantly enhances its piezoelectric effect, which can improve the piezoelectric properties of ZnO. Compared with Mg-doped ZnO in previous studies, the piezoelectric coefficient is increased by 2.6 times. Therefore, the Li doping method is selected in this paper to fabricate sensitive thin films.

## 4. Preparation and Testing of Three Li-Doped ZnO Ultra-Thin Cantilever Beam Acceleration Sensors

Based on theoretical analysis, this design adopts P-type <100> crystal-oriented double-polished silicon wafer with a thickness of 400 µm and a resistivity of 0.01~0.02 Ω.cm. The designed film size underwent stress matching design and piezoelectric film fabrication. Through the characterization of piezoelectric thin films, the test results match the first-principles results. In order to facilitate the testing of the piezoelectric coefficient, a single crystal silicon wafer was selected before the production of ZnO thin films. SiO_2_/Si_3_N_4_ thin film was produced, and Pt film was used as the lower electrode of the piezoelectric thin film. Based on the first-principles calculation results, due to the limitation of sputtering target concentration, Li/ZnO targets with different doping ratios of 0% and 3% were selected for the production of ZnO thin films using magnetron sputtering. XPS characterization and piezoelectric coefficient testing were performed on the ZnO thin films. In order to increase the thickness of the ZnO films and ensure that the growth conditions of the ZnO films were the same as those in the sensor, SiO_2_/Si with a Pt/Ti composite electrode was selected as the substrate. Li-doped ZnO was selected as the sputtering target material, and Li_2_CO_3_ with different molar ratios was added to the ZnO analytical pure raw material, which was then fired at high temperature and high pressure. The doping concentration of the Li-doped ZnO target material was 3%. In order to reduce testing errors, the sputtering power was increased to 150 W to increase the thickness of the ZnO film. In order to ensure that the growth conditions of the ZnO thin films were the same as those in subsequent sensors, SiO_2_/Si with Pt/Ti composite electrodes was selected as the substrate. The flow rates of O_2_ and Ar were 20 sccm and 20 sccm, and the sputtering time was 30 min. Other parameters are shown in Table 3.

**Figure 10:**
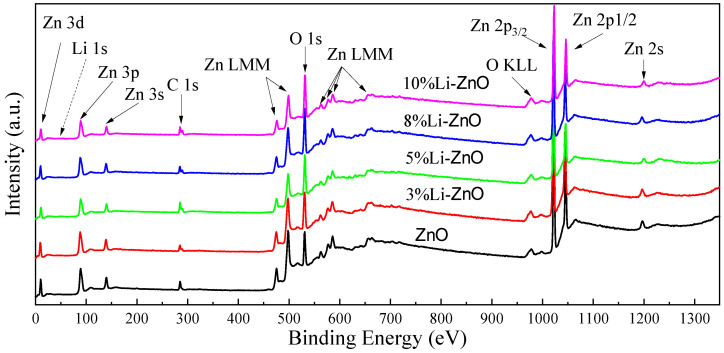
Full XPS spectra of undoped and Li-doped ZnO thin films.

We performed X-ray photoelectron spectroscopy (XPS) analysis on the experimental samples to analyze the effect of changes in Li doping concentration on the atomic content of Zn, O, and Li in Zn_1−x_Li_x_O, This is shown in Figure 10.

**Figure 11:**
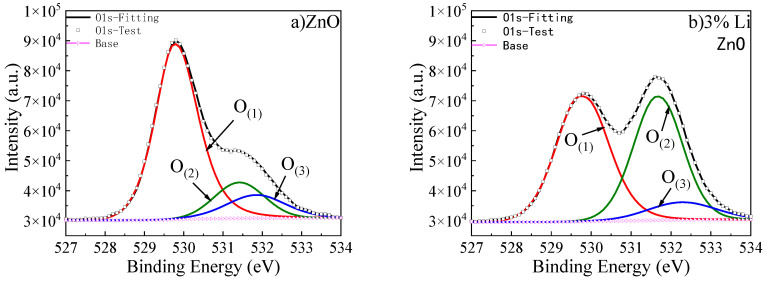
Deconvolution spectra of ZnO thin films with O1s and different Li doping concentrations. (**a**) Undoped; (**b**) 3% Li-doped.

Gaussian fitting was used to deconvolve the O1s peak XPS high-resolution spectra of undoped ZnO and ZnO with different Li doping concentrations, and the results are shown in Figure 11.

In ZnO thin films, two situations occur when Li is doped into ZnO. (1) Li atoms, as interstitial atoms, lose one electron and are located in the interstitial position of the lattice. At this point, in order to exhibit electrical neutrality, oxygen atoms gain two electrons, which carry two negative charges relative to the original lattice O, and act as metallic oxygen in the interstitial position. (2) Li replaces the position of Zn, losing one electron and forming Li^+^. Compared to Zn^2+^, Lizn^−^ carries a negative charge. To ensure charge conservation, O vacancies are generated that carry two positive charges relative to the original lattice O. At the same time, this is accompanied by the appearance of surface chemisorbed oxygen. Therefore, the O element can exist in three forms, represented by the following symbols: metallic oxygen—O_(1)_; oxygen vacancy—O_(2)_; surface chemisorbed oxygen—O_(3)_.

This is shown in Figure 11, when the Li element is introduced, the content of O_(1)_ decreases significantly. As the Li doping concentration increases, the rate of decrease in O_(1)_ content slows down, while O_(2)_ and O_(3)_ show an upward trend. This indicates that Li mainly enters ZnO as substitutional LiZn. When the Li doping concentration reaches 5%, the upward trend of O_(2)_ content slows down, indicating that the substitutional impurity of LiZn tends to saturate. As the Li doping concentration increases, the LiZn impurity content approaches saturation, while the Li doping amount continues to increase. Therefore, when the doping concentration exceeds 5%, the interstitial Li content will gradually increase, leading to an increase in metal oxygen content. With the introduction of Li doping, oxygen vacancies appear, and their defect positions capture surface chemisorbed oxygen, resulting in an increase in surface chemisorbed oxygen content.

**Figure 12:**
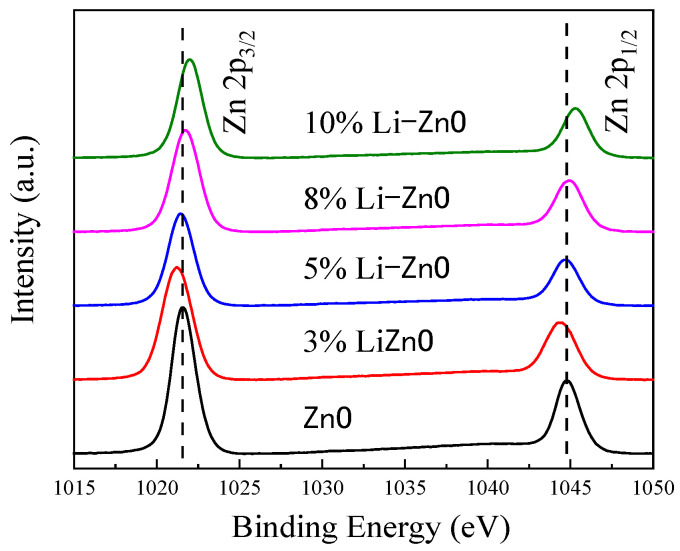
ZnO thin films with different Li doping concentrations.

From the Zn 2p curve Figure 12, it can be seen that in the undoped state, two asymmetric double peaks appear at binding energies of 1021.58 eV and 1044.71 eV, with a difference in binding energy ΔE of 23.13 eV. This is due to spin splitting of the Zn 2p electron orbitals, and the peak binding energy and the difference in binding energy are consistent with relevant reports. At the same time, it was observed that when the Li doping concentration was 3%, the binding energies of Zn 2p_1/2_ and Zn 2p_3/2_ both weakened. As the doping concentration increases, the binding energy of Zn 2p increases. When the Li doping concentration reaches 5%, the binding energy of Zn 2p is similar to that of undoped ZnO. When the doping concentration reaches 10%, the binding energy shifts to the maximum, with Zn 2p_3/2_ at 1045.22 eV and Zn 2p_1/2_ at 1021.96 eV. When the Li element is introduced, Li acts as a substitute atom—Lizn—and oxygen vacancies appear, causing an increase in the outer electron cloud of Zn around the two electrons of the oxygen vacancy defect, acting as a barrier. At this point, the content of oxygen vacancies with two positive charges increases, leading to an increase in the outer electron cloud density of the surrounding Zn. According to the definition of binding energy (the energy required to move an electron to infinity), the binding energy decreases and the spectral line shifts to the left. When the doping amount of Li element continues to increase and reaches 5%, Liz tends to saturate, and the content of Li in interstitial atoms increases. Compared with the metal oxygen with two negative charges, the content of Li increases, causing a decrease in the electron cloud density of the surrounding Zn outer layer, weakening the shielding effect, increasing the binding energy of Zn 2p, and shifting the spectral line to the right. The first-principles calculations of the test results are consistent, and Li-doped ZnO can effectively increase the piezoelectric properties of ZnO.

**Figure 13:**
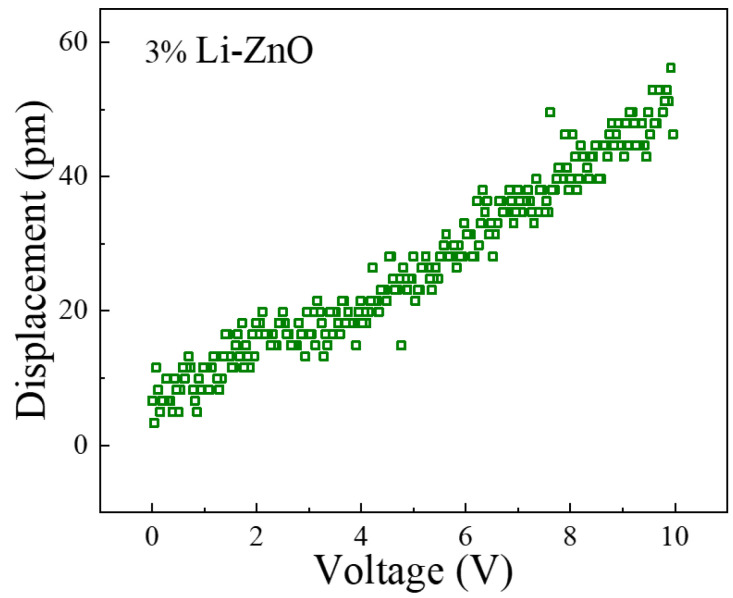
PFM test data of 3% Li-doped ZnO piezoelectric thin film.

The PFM test data for the 3% Li-doped ZnO piezoelectric thin film is shown in Figure 13, with excitation voltage ranging from 0 to 10 V. As shown in the figure, the d33 piezoelectric coefficient of the 3% Li-doped ZnO piezoelectric film has a value of 4.42 pm/V. Based on the structural dimensions used in this design, the piezoelectric coefficient has a value of 2.335 C/m^2^, which is basically consistent with the first-principles calculation results.

If the thickness of the cantilever beam is too large, it will reduce the sensitivity of the sensor. If the thickness is too thin, it will reduce the support effect of the cantilever beam. At the same time, in order to increase the piezoelectric performance of the piezoelectric layer, based on previous research results, the thickness is set to 0.2 μm, and combined with stress theory design, the structural dimensions of each layer are set as shown in Table 4.

**Figure 14:**
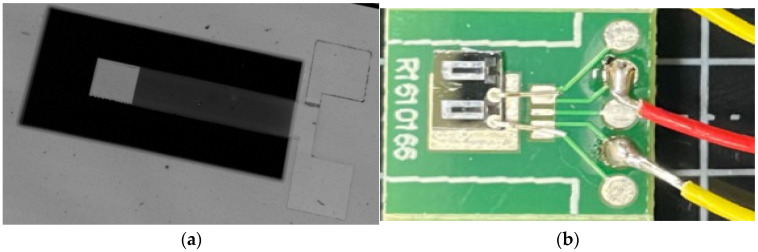
Sample picture: (**a**) Chip diagram; (**b**) Test sample diagram.

Using MEMs technology, SiO_2_ thin-film production, Si_3_N_4_ thin-film production, sacrificial layer mask production, and sacrificial layer production are carried out successively. The removal of SiO_2_/Si_3_N_4_, the production of low-stress multi-layer composite films, the production of multi-layer composite cantilever beam structures, and the release of cantilever beam structures are achieved. An acceleration sensor has been produced, and the sample produced is shown in Figure 14. Figure 14a consists of SiO_2_, Si_3_N_4_, the Pt lower electrode, the Li-doped ZnO thin film, and the Pt upper electrode. In Figure 14b, the fabricated chip is bonded to the circuit board, and wire bonding is performed using platinum metal sintering technology. We conducted frequency and force sensitivity tests on the produced ultrafine force sensor and used a frequency scanner to test the resonant frequency of the sensor. Due to the simulation result of the resonant frequency of the sensor being 4.5 kHz, the sweep frequency range was set to 1~10 kHz and refined between 3.6 kHz and 4.6 kHz. The test results are shown in Figure 13.

**Figure 15:**
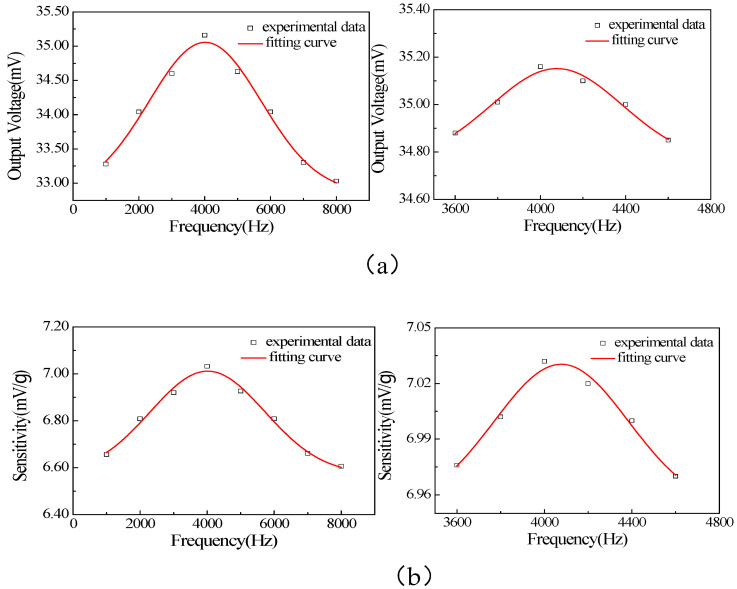
Test results of pattern products: (**a**) relationship curve between frequency and output voltage; (**b**) sensitivity.

By using heterogeneous thin-film internal stress matching technology, the internal stress of the suspended beam film can be effectively reduced, the warpage of the film can be lowered, and the flatness and toughness of the film can be improved. For the above structure, sample production was carried out. The samples in the preparation stage are shown in Figure 14. The sample was subjected to acceleration signal sweep frequency testing and sensitivity testing. After testing, its resonant frequency was 4 kHz and sensitivity was 7.04 mV/g. The test results are shown in Figure 15. Compared with previous research results, it was found that using ultra-thin suspension film and Li-doped ZnO as a piezoelectric sensitive thin film can effectively improve the sensitivity of the accelerometer.

## 5. Conclusions

A detailed analysis was conducted on the motion mechanism of ultra-thin heterogeneous cantilever beams, and the relationship between the size of the cantilever beam and strain effects was determined. First-principles calculations were conducted on the energy band, density of states, charge density, and piezoelectric coefficient of Li-doped ZnO. According to our calculations, as the Li doping ratio increases from 0 to 10%, the bandgap width of ZnO material increases from 0.74 to 1.21 eV. The results of the density of states and partial density of states analyses indicate that the increase in band gap is due to the movement of Zn-3d states towards the high-energy end, and the piezoelectric coefficient of the material increases from 2.07 to 3.3 C/m^2^. Based on theoretical calculations, we created a cantilever beam sensor structure with relatively balanced internal stress. The frequency and sensitivity characteristics of the produced ultrafine force sensor were tested. The test results showed that the resonant frequency of the prepared sensor was 4 KHz, the sensitivity was 7.04 mV/g, and it had good linearity. New insights can be obtained via the development of acceleration, vibration sensors, and photoanodes.

## Figures and Tables

**Table 1:** Comparison of first-principles models of different materials and sensitivity of prepared acceleration sensors.

Year	Materials	Preparation Method	First-Principles Model	Sensitivity
2020	ZnO	Magnetron sputtering	Primitive cell expansion 5 × 2 × 1	0.45 mV/g
2021	AlN/Sc0.2Al0.8N	Magnetron sputtering	Primitive cell expansion 5 × 2 × 1	0.84 mV/g
2022	AlN	Magnetron sputtering	Primitive cell expansion 9 × 9 × 6	1.553 mV/g
2022	PZT	Sol–gel	Primitive cell expansion 2 × 2 × 2	28.14 mV/g
2023	PZT	Aerosol deposition	Primitive cell expansion 2 × 2 × 2	4.96 mV/g

**Table 2:** Li-doped ZnO lattice constant table.

	0%	2.5%	5%	7.5%	10%
a	0.3249	0.3251	0.3251	0.3251	0.3252
c	0.5206	0.5134	0.5131	0.5126	0.5122

**Table 3:** Sputtering parameters of ZnO thin films.

Sputtering Power (W)	O_2_: Ar (sccm)	Ultimate Pressure (Pa)	Sputtering Pressure (Pa)	Sputtering Time (min)	Sputtering Temperature (°C)
150	20:20	1.0 × 10^−3^	1.0	30	indoor temperature

**Table 4:** Dimensions of each layer structure of acceleration sensor.

Designation	Materials	Parameter	Parameter Value (μm)
Structural layer	SiO_2_ Si_3_N_4_	ls	1400
		ws	300
		hs	2
Bottom electrode	Ti	lTi	1400
		wTi	300
		hTi	0.05
Bottom electrode/top electrode	Pt	lPt	1400
		wPt	300
		hPt	0.15
Piezoelectric layer	Li/ZnO	lZn	1400
		wZn	300
		hZn	0.2
Mass block	Poly-Si	lm	300
		wm	300
		hm	1

## Data Availability

The original contributions presented in this study are included in the article. Further inquiries can be directed to the corresponding author.
